# Major histocompatibility complex associations of ankylosing spondylitis are complex and involve further epistasis with *ERAP1*

**DOI:** 10.1038/ncomms8146

**Published:** 2015-05-21

**Authors:** Adrian Cortes, Sara L. Pulit, Paul J. Leo, Jenny J. Pointon, Philip C. Robinson, Michael H. Weisman, Michael Ward, Lianne S. Gensler, Xiaodong Zhou, Henri-Jean Garchon, Gilles Chiocchia, Johannes Nossent, Benedicte A. Lie, Øystein Førre, Jaakko Tuomilehto, Kari Laiho, Linda A. Bradbury, Dirk Elewaut, Ruben Burgos-Vargas, Simon Stebbings, Louise Appleton, Claire Farrah, Jonathan Lau, Nigil Haroon, Juan Mulero, Francisco J. Blanco, Miguel A. Gonzalez-Gay, C Lopez-Larrea, Paul Bowness, Karl Gaffney, Hill Gaston, Dafna D. Gladman, Proton Rahman, Walter P. Maksymowych, J. Bart A. Crusius, Irene E. van der Horst-Bruinsma, Raphael Valle-Oñate, Consuelo Romero-Sánchez, Inger Myrnes Hansen, Fernando M. Pimentel-Santos, Robert D. Inman, Javier Martin, Maxime Breban, Bryan Paul Wordsworth, John D. Reveille, David M. Evans, Paul I.W. de Bakker, Matthew A. Brown

**Affiliations:** 1 University of Queensland Diamantina Institute, Princess Alexandra Hospital, Brisbane 4102, Australia; 2 Department of Medical Genetics, Center for Molecular Medicine, University Medical Center Utrecht, Utrecht 3584, The Netherlands; 3 NIHR Oxford Musculoskeletal Biomedical Research Unit, Nuffield Orthopaedic Centre, Headington, Oxford OX3 7LD, UK; 4 Department of Medicine/Rheumatology, Cedars-Sinai Medical Center, Los Angeles, California 90048, USA; 5 Intramural Research Program, National Institute of Arthritis and Musculoskeletal and Skin Diseases, National Institutes of Health, Bethesda, Maryland 20892-1468, USA; 6 Department of Medicine/Rheumatology, University of California, San Francisco, California 94143, USA; 7 Department of Rheumatology and Clinical Immunogenetics, University of Texas Health Science Center at Houston, Houston, Texas 77030, USA; 8 INSERM UMR 1173, Université de Versailles Saint Quentin en Yvelines, Laboratoire d’excellence Inflamex, Saint-Quentn-En-Yvelines 78180, France; 9 Genetics Division, Hôpital Ambroise Paré, AP-HP, and Université de Versailles Saint Quentin en Yvelines, Boulogne-Billancourt 78180, France; 10 School of Medicine, University of Western Australia, Perth, WA 6009, Australia; 11 Department of Rheumatology, Sir Charles Gairdner Hospital, Perth, WA 6009, Australia; 12 Department of Medical Genetics, University of Oslo and Oslo University Hospital, Oslo 0310, Norway; 13 Department of Immunology, Oslo University Hospital, Oslo 0310, Norway; 14 Department of Rheumatology, Oslo University Hospital, and University of Oslo, Oslo 0310, Norway; 15 Department of Chronic Disease Prevention, National Institute for Health and Welfare, 00271 Helsinki, Finland; 16 Centre for Vascular Prevention, Danube-University Krems, 3500 Krems, Austria; 17 Diabetes Research Group, King Abdulaziz University, 21589 Jeddah, Saudi Arabia; 18 Department of Medicine, Paijat-Hame Central Hospital, Lahti, Finland; 19 Department of Rheumatology, Gent University Hospital, Gent 9052, Belgium; 20 VIB Inflammation Research Center, Ghent University, Gent 9052, Belgium; 21 Department of Rheumatology, Hospital General de Mexico and Universidad Nacional Autonoma de Mexico, Mexico City 06726, Mexico; 22 Department of Medicine, Dunedin School of Medicine, University of Otago, Dunedin 9016, New Zealand; 23 Division of Rheumatology, Toronto Western Hospital, University of Toronto, Toronto M5T2S8, Canada; 24 Department of Rheumatology, Hospital Puerta de Hierro, Madrid 28222, Spain; 25 Department of Rheumatology, Complejo Hospitalario La Coruña, INIBIC, La Coruña 15006, Spain; 26 Department of Rheumatology, Hospital Marques de Valcecillas, IFIMAV, Santander 39008, Spain; 27 Department of Immunology, Hospital Universitario Central de Asturias, Oviedo 33011, Spain; 28 Fundación Renal ‘Iñigo Álvarez de Toledo’, Madrid 33011, Spain; 29 Department of Rheumatology, Norfolk and Norwich University Hospital, Norwich NR4 7UY, UK; 30 Department of Medicine, University of Cambridge, Addenbrookes Hospital, Cambridge CB2 0SP, UK; 31 Division of Rheumatology, University of Toronto, Toronto, ON M4N 3M5, Canada; 32 Toronto Western Research Institute, Toronto ON M4N 3M5, Canada; 33 Psoriatic Arthritis Program, University Health Network, Toronto ON M4N 3M5, Canada; 34 Memorial University of Newfoundland, Newfoundland NL A1B 3X9, Canada; 35 Department of Medicine, University of Alberta, Alberta T6G 2R7, Canada; 36 Department of Medical Microbiology and Infection Control, Laboratory of Immunogenetics, VU University Medical Center, Amsterdam 1081 BT, The Netherlands; 37 Department of Rheumatology, VU University Medical Centre, Amsterdam 1081 BT, Netherlands; 38 Spondyloarthropaty Group-Division of Rheumatology, Hospital Militar Central/Universidad de La Sabana, Transversal 3 # 49-00, Bogotá, NA, Colombia; 39 Helgelandssykehuset, 8613 Mo i Rana, Norway; 40 Chronic Diseases Research Centre (CEDOC), Faculdade de Ciências Médicas, Universidade Nova de Lisboa, Lisboa 1169-056, Portugal; 41 Instituto de Parasitología y Biomedicina López-Neyra, Consejo Superior de Investigaciones–Científicas, Granada 18100, Spain; 42 Division of Rheumatology, Hôpital Ambroise Paré, AP-HP, and Université de Versailles Saint Quentin en Yvelines, Boulogne-Billancourt 92100, France; 43 MRC Integrative Epidemiology Unit, University of Bristol, Bristol, UK; 44 School of Social and Community Medicine, University of Bristol, Bristol, UK; 45 Department of Epidemiology, Julius Center for Health Sciences and Primary Care, University Medical Center Utrecht, Utrecht, The Netherlands

## Abstract

Ankylosing spondylitis (AS) is a common, highly heritable, inflammatory arthritis for which HLA-B*27 is the major genetic risk factor, although its role in the aetiology of AS remains elusive. To better understand the genetic basis of the MHC susceptibility loci, we genotyped 7,264 MHC SNPs in 22,647 AS cases and controls of European descent. We impute SNPs, classical HLA alleles and amino-acid residues within HLA proteins, and tested these for association to AS status. Here we show that in addition to effects due to HLA-B*27 alleles, several other *HLA-B* alleles also affect susceptibility. After controlling for the associated haplotypes in *HLA-B*, we observe independent associations with variants in the *HLA-A*, *HLA-DPB1* and *HLA-DRB1* loci. We also demonstrate that the *ERAP1* SNP rs30187 association is not restricted only to carriers of HLA-B*27 but also found in HLA-B*40:01 carriers independently of HLA-B*27 genotype.

## Abstract (web summary)

Ankylosing spondylitis is a common, highly inheritable inflammatory arthritis with poorly understood biology. Here Brown, Cortes and colleagues use fine mapping of the major histocompatibility complex and identify novel associations, and identify other HLA alleles that like HLA-B27 interact with ERAP1 variants to influence disease risk.

## Graphical abstract

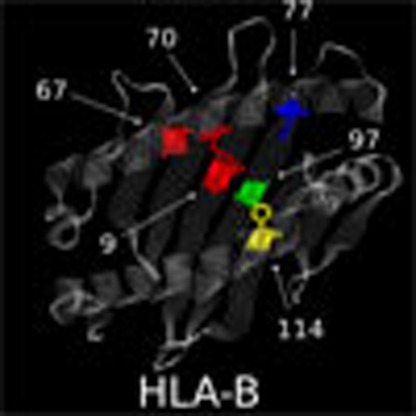

Ankylosing spondylitis (AS) is a common, highly heritable^1^, inflammatory arthritis for which HLA-B*27 is the major genetic risk factor. To better understand the genetic basis of the major histocompatibility complex (MHC) susceptibility loci, we genotyped 7,264 MHC single-nucleotide polymorphisms (SNPs) in 9,069 AS cases and 13,578 population controls of European descent using the Illumina Immunochip microarray. In addition to extremely strong effects due to HLA-B*27:02 and B*27:05, several other *HLA-B* alleles (B*07:02, B*13:02, B*40:01, B*40:02, B*47:01, B*51:01 and B*57:01) also affect susceptibility to AS. *HLA-B*-independent associations were demonstrated with variants in the *HLA-A*, *HLA-DPB1* and *HLA-DRB1* loci. We also demonstrate that the *ERAP1* SNP rs30187 association is not restricted only to carriers of HLA-B*27 but also found in HLA-B*40:01 carriers independently of the HLA-B*27 genotype. The presence of associations in both HLA class I and II loci might reflect effects on antigen presentation to both CD4^+^ and CD8^+^ T lymphocytes in the pathogenesis of AS.

While the classical HLA-B*27 allele is found in over 85% of AS patients^2^,^3^,^4^, it is clearly not sufficient alone to cause disease, with only 1–5% of HLA-B*27 carriers developing the disease. From epidemiological data, it is evident that susceptibility to AS is affected by other genes within and outside the MHC^1^. Indeed, 26 risk loci outside the MHC have now been identified by genome-wide association studies^5^,^6^,^7^,^8^.

The biological mechanism(s) by which HLA-B27 confers risk of disease remains elusive. The main hypotheses regarding this mechanism can be divided into canonical mechanisms based on the known function of HLA-B27 within the adaptive immune system, and non-canonical mechanisms related to unusual properties of HLA-B27, notably its propensity to dimerise or misfold. Suggested canonical mechanisms propose either that HLA-B27 is uniquely capable of presenting particular peptide(s) found at sites of inflammation in AS to cytotoxic T lymphocytes (the arthritogenic peptide hypothesis)^9^ or that HLA-B27 is associated with reduced gut mucosal immunity, leading to migration of enteric bacteria across the intestinal mucosa, driving the production of the pro-inflammatory cytokine interleukin (IL)-23 and development of AS (the mucosal immunodeficiency hypothesis)^10^,^11^. Both these theories place antigenic peptide presentation and handling as critical steps in the pathogenesis of AS. One of the first non-MHC susceptibility loci to be identified in AS was endoplasmic reticulum aminopeptidase 1 (*ERAP1*)^5^, the main function of which is to trim peptides in the endoplasmic reticulum (ER) to optimal length for binding to MHC class I molecules on antigen-presenting cells for subsequent interaction with CD8^+^ T cells^12^,^13^. Moreover, this association is so far uniquely found in HLA-B*27-positive disease^7^.

HLA-B27 has an unusual property of forming homodimers through disulphide bonding of the unpaired cysteine residue at position 67 (ref. 14). It has been proposed that these homodimers may cause AS through abnormal presentation of peptides or by facilitating ‘abnormal’ interaction with natural killer cells^15^. Apart from HLA-B*27, the subtypes of the alleles HLA-B*14, HLA-B*15, HLA-B*38, HLA-B*39 and *HLA*-B*75 encode a cysteine residue at position 67 but of these there is only evidence that HLA-B*14 may be AS associated^16^,^17^. It is also unclear if these other non-HLA-B27 Cys67 variants can form homodimers. In addition, Cys67 is found on all HLA-B27 subtypes, including the subtypes HLA-B*27:06 and HLA-B*27:09, which are not AS associated^18^,^19^. A further hypothesis suggests that abnormal folding of the HLA-B27 molecule during assembly results in ER stress and activation of the unfolded protein response^20^,^21^. ER stress is evident in the HLA-B*27-transgenic rat model of AS and correlates with production of IL-23 (ref. 21), but has not been demonstrated in HLA-B*27-positive patients^22^,^23^,^24^.

While non-B27 HLA associations have been reported, notably with *HLA-B40* (refs 25, 26, 27) and HLA-A*02 (ref. 8), most have not been definitive or replicated in independent studies. In this study, we analyse the associations of AS across the MHC aiming to identify functional and potentially causal variants using a large, previously reported, panel of cases and controls of European ancestry^8^. Here we extend on our primary analysis of this cohort by fine mapping the MHC region with imputation of SNPs, MHC class I and II classical alleles, and amino-acid residues within the classical HLA proteins^28^. In addition to HLA-B27, we identify further HLA-B and other HLA class I and II alleles associated with AS, and demonstrate that HLA-B40 in addition to HLA-B27 interacts with *ERAP1* to cause disease. This implicates both CD4 and CD8 lymphocytes in AS pathogenesis and suggests that HLA-B40 and HLA-B27 operate by similar mechanisms to induce the disease.

## Results

### HLA-B susceptibility alleles

**Figure 1:**
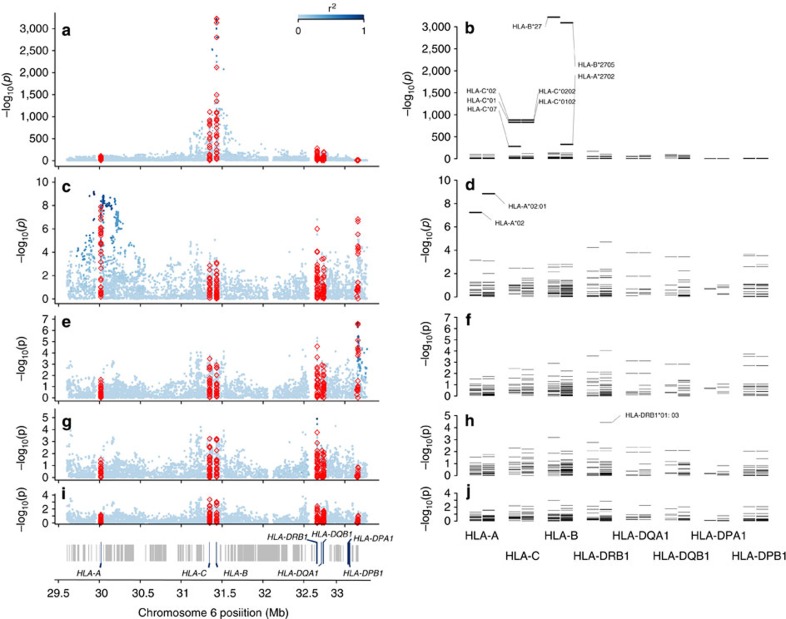
Association results with ankylosing spondylitis susceptibility in the MHC. Omnibus SNP and amino-acid association tests are shown in **a**, **c**, **e**, **g** and **i**, and classical allele association tests with two- and four-digit resolution in **b**, **d**, **f**, **h** and **j**. The strongest association was found with positions in the polymorphic nucleotide rs41558317 and in the polymorphic amino acid 97 of HLA-B (**a**), and with the HLA-B*27 allele (**b**). Controlling for the effect of *HLA-B* susceptibility alleles, an independent association was observed with SNPs and amino-acid position in the *HLA-A* locus (**c**) corresponding to the HLA-A*02:01 allele (**d**). Further conditioning on *HLA-A* and *HLA-B* loci an independent association with SNPs and amino-acid positions in the *HLA-DPB1* locus was evident (**e**); no *HLA-DPB1* classical allele was significant at the same magnitude as the SNPs and amino-acid positions (**f**). Further controlling for the effect of variation in the *HLA-DPB1* locus association was observed with SNPs in the *HLA-DRB1* locus (**g**, **h**). SNP association tests are shown in blue circles, colour coded by linkage disequilibrium from the SNP with the strongest association. Amino-acid position tests are shown as red triangles. Classical allele tests are shown as bars for two- and four-digit imputation resolution.

At the *HLA-B* locus, 38 classical alleles at four-digit resolution were imputed. All SNP, HLA and amino-acid association *P*-values were determined by logistic regression. As expected, the two common HLA-B*27 alleles in the European population, B*27:02 (odds ratio (OR)=43; *P*=1.07 × 10^−122^) and B*27:05 (OR=62; *P*<10^−321^), were the most significantly associated with disease risk (Fig. 1a–b; Tables 1 and 2). Controlling for the effect of the two B*27 alleles, we identified the protective alleles HLA-B*07:02 (OR=0.82; *P*=5.04 × 10^−6^) and HLA-B*57:01 (OR=0.75; *P*=5.13 × 10^−4^; Table 2). Moderate association was also observed, sequentially, with the risk alleles HLA-B*51:01 (OR=1.33; *P*=2.14 × 10^−3^), HLA-B*47:01 (OR=2.35; *P*=2.25 × 10^−3^), HLA-B*40:02 (OR=1.59; *P*=4.65 × 10^−3^), HLA-B*13:02 (OR=1.43; *P*=4.29 × 10^−3^) and HLA-B*40:01 (OR=1.22; *P*=4.93 × 10^−3^). No evidence of further susceptibility alleles was observed after controlling for the risk and protective alleles identified above (*P*>0.05; Fig. 1d). The *HLA-B* associations were similar in both HLA-B*27-positive and HLA-B*27-negative restricted analyses (Supplementary Tables 1–6).

### Non-*HLA-B* susceptibility loci in the MHC

To assess whether other MHC loci affect disease susceptibility independently from the *HLA-B* locus, we performed additional conditional analyses. Adjusting for the *HLA-B* susceptibility alleles identified, we observed an association signal with SNPs in the *HLA-A* locus (rs2975033; OR=1.22; *P*=6.16 × 10^−10^) and with the classical allele HLA-A*02:01 (OR=1.22; *P*=1.41 × 10^−9^; Fig. 1c–d). The risk allele ‘A’ of rs2975033 was in near perfect linkage disequilibrium with the risk allele HLA-A*02:01 (*r*^2^=0.97).

Further controlling for the effect of the susceptibility SNP rs2975033 in *HLA-A* revealed an independent signal with SNPs (rs1126513; OR=1.21; *P*=2.46 × 10^−7^) in the class II locus *HLA-DPB1* (Fig. 1e); no association of similar strength to those seen with SNPs (*P*>10^−5^) were observed with classical *HLA-DPB1* alleles (Fig. 1f). After controlling for the effect of the SNP rs1126513 in *HLA-DPB1*, we observed an association with the SNP rs17885388 (OR=1.16; *P*=1.27 × 10^−5^) in the *HLA-DRB1* locus, and a similar level of significance was also observed with the class II allele HLA-DRB1*01:03 (*P*=3.78 × 10^−5^). No further associations were observed after controlling for all identified effects (*P*>5 × 10^−5^; Fig. 1i–j).

### Association signals and amino-acid positions in HLA proteins

**Figure 2:**
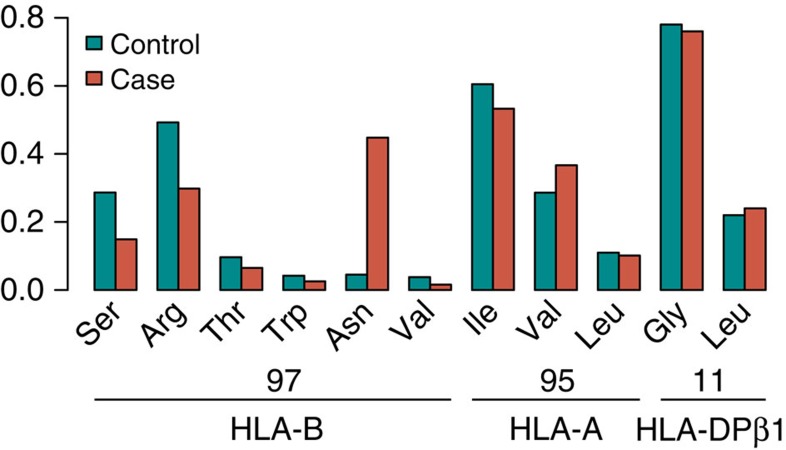


**Figure 3:**
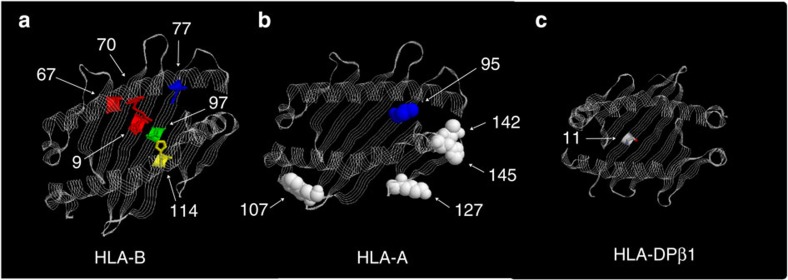
Three-dimensional models for the HLA-B, HLA-A and HLA-DPβ1 proteins. Three-dimensional models for the (**a**) HLA-B, (**b**) HLA-A and (**c**) HLA-DPB1 proteins. These structures are based on Protein Data Bank entries 3LV3, 3UTQ and 3LQZ, respectively, with a direct view to the peptide-binding groove.

We observed disease-associated alleles at MHC class I and II loci. Classical alleles at these loci determine the amino-acid sequence of the respective HLA proteins, which could in turn influence the specificity of the peptides presented to CD8^+^ and CD4^+^ T lymphocytes. We, therefore, analysed the polymorphic amino-acid residues at these proteins to assess their effect in disease susceptibility. In this analysis, the strongest association was observed for amino-acid position 97 in HLA-B (omnibus *P*<10^−3221^; Table 1; Fig. 1a–b). In addition, through conditional analysis, we found that the association at the HLA-B*27 allele, and other HLA-B*27-associated polymorphisms, was explained by position 97 while the reverse was not true (Supplementary Table 7). This polymorphic position carries as many as six different amino-acid residues in the population (Fig. 2), each conferring a different degree of risk (or protection) to disease, consistent with the analysis of *HLA-B* alleles mentioned above (Table 2). Position 97 lies in the floor of the HLA-B peptide-binding groove (Fig. 3), located in the C/F pocket, also referred as the C-terminal pocket, which anchors the side chain of the C-terminal peptide residue^29^. Asparagine at position 97 is uniquely observed in HLA-B*27 alleles. Threonine at position 97 (predominantly found in HLA-B*51 alleles) was also found to increase disease risk (OR=1.12; *P*=4.50 × 10^−3^); serine (found in HLA-B*07 and *08 alleles) decreased risk of disease (OR=0.86; *P*=5.2 × 10^−8^); and valine (found in HLA-B*57 alleles) was also protective (OR=0.68; *P*=1.4 × 10^−8^; Table 3).

Strong associations were also observed with the amino-acid positions 70, 114, 77 and 67 of HLA-B but these signals were strongly attenuated after conditioning on amino-acid position 97. In contrast, none of these positions could explain the association at position 97. In particular, there was little evidence of association at position 67 (that is, the position where disulphide bonding of unlinked cysteine residues might occur) after conditioning on position 97 (*P*-value=0.04; Supplementary Table 8).

The most strongly associated amino acid of the HLA-A molecule, after conditioning on associated HLA-B alleles, was amino acid valine at position 95 (*P*=3.70 × 10^−9^). The association with this amino acid was statistically equivalent with that observed with the SNP rs2975033 and with the classical allele HLA-A*02:01. This amino acid is positioned within the binding site of HLA-A (Fig. 3).

Independent associations were observed at the two class II loci *HLA-DPB1* and *HLA-DRB1*, and these were highly correlated with polymorphic amino acids in the peptide-binding site of these molecules (Fig. 3). At the *HLA-DPB1* locus, rs1126513 showed the strongest association and the risk allele for rs1126513 was perfectly correlated with the presence of leucine at position 11 of the HLA-DPB1 molecule (position 11; OR=1.21; *P*-value=2.46 × 10^−7^). At the *HLA-DRB1* locus, the strongest association with an amino acid was observed with aspartic acid at position 70 (OR=1.16; *P*-value=3.44 × 10^−5^); due to linkage disequilibrium this association was statistical equivalent to the one observed with the SNP rs17885388.

### Gene–gene interactions and susceptibility loci

**Figure 4:**
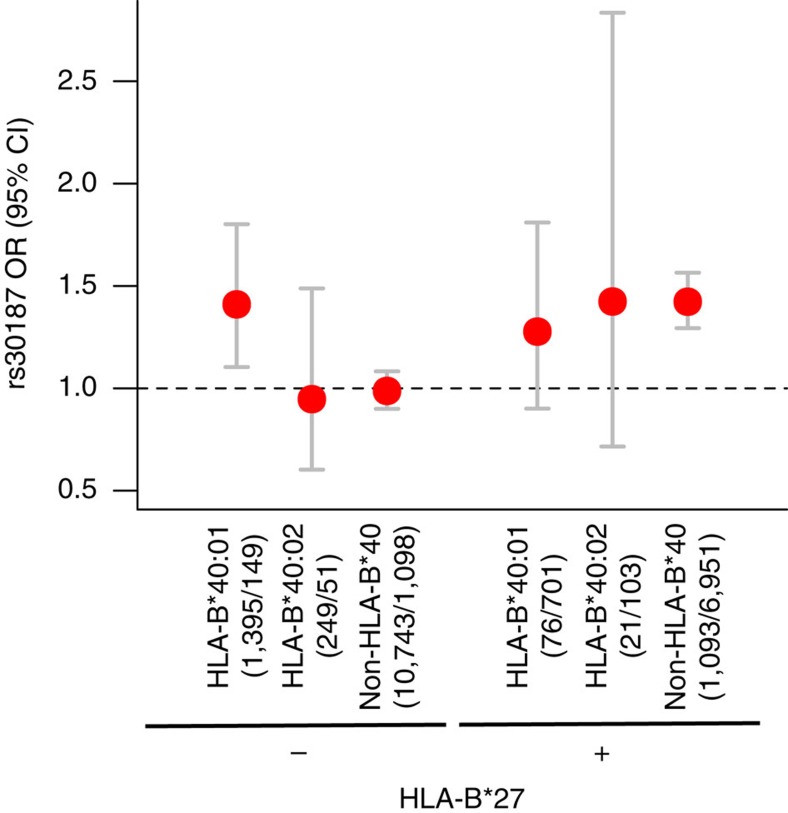
Interaction between *ERAP1* and *HLA-B* susceptibility alleles. For each stratified group, effect size for the *ERAP1* variant rs30187 is given. Error bars represent 95% confidence intervals. Number of samples in each group (controls/cases) is given below the HLA-B*40 genotype.

We have previously observed that the association with the variant rs30187 in the *ERAP1* locus is restricted to HLA-B*27-positive subjects, consistent with an epistatic interaction between these two loci^7^. Here we investigated the possibility of interaction between the other *HLA-B* susceptibility alleles and the variant rs30187. When testing for interaction with the HLA-B*40 alleles, we found that rs30187-T increased the risk of disease in the strata where HLA-B*27 was present, as previously shown, or when HLA-B*40:01 was present in the absence of HLA-B*27 (OR=1.41; *P*=5.81 × 10^−3^); rs30187 had no effect on disease susceptibility when both HLA-B*27 and HLA-B*40 alleles were absent or in the non-HLA-B*27/HLA-B*40:02 stratum (Fig. 4). No evidence of interaction was observed between rs30187 and the other *HLA-B* susceptibility alleles. This suggests that the rs30187 variant interacts with the HLA-B*40:01 allele; although no evidence to support an interaction was observed with HLA-B*40:02, the study had low power to detect such an effect. There was no evidence of interaction between either of the HLA-B*40 alleles and any of the other independently associated susceptibility SNPs in the loci encoding the aminopeptidases *ERAP1*, *ERAP2* and *NPEPPS* (*P*>0.1).

We then examined whether our data supported a model where the HLA-B*27 and *HLA*-B*40 alleles increased disease susceptibility beyond their inferred independent effects, as previously reported^30^. No support for an interaction between these alleles was observed in this data set (Supplementary Table 1).

## Discussion

Independently of the expected *HLA-B* associations, this study demonstrates that both HLA-B*40:01 and -B*40:02 are disease associated alleles, and identified three further *HLA-B* risk alleles, HLA-B*51:01, B*47:01 and B*13:02. The allele HLA-B*51:01 is also the major genetic risk factor for Behçet’s disease^31^, a seronegative disease complicated by sacroiliitis resembling AS in up to 10% of cases^32^. In addition to the seven *HLA-B* risk alleles, we identified two protective alleles at this locus, HLA-B*07:02 and HLA-B*57:01. Interestingly, in the HLA-B*27-transgenic rat model of AS, the HLA-B*27-negative control carries the HLA-B*07 allele, and does not develop disease, consistent with the protective effect of this allele in humans^33^. It has recently been shown in *HLA-B7/B27* co-expressing mice that there is partial negative selection of *HLA-B27+* T cells in the course of defining the immunodominant response to influenza infection^34^. Further, in *Erap*-deficient, influenza-infected *HLA-B27*-positive mice, there was a marked reduction in presentation of the HLA-B27 immunodominant epitope, and T-cell immunity to that epitope, presumed to be because the HLA-B27-related immunodominant flu epitope requires cleavage by Erap to be presented by HLA-B27. In contrast, in *HLA-B7*-transgenic mice, *Erap* deficiency had no effect on presentation of the HLA-B7 immunodominant epitope or the corresponding T-cell response to it, suggesting that it does not require Erap cleavage for presentation^35^. This provides a potential mechanism to explain the genetic effects observed in humans with AS, with ERAP1 loss of function protecting against HLA-B27-associated AS, but having no effect in HLA-B7 carriers, where an HLA-B7 protective association is observed.

Outside the *HLA-B* locus, we identified three independent significant signals associated with AS; one was in the HLA class I locus *HLA-A*, and one each in the HLA class II loci *HLA-DPB1* and *HLA-DRB1*. The association in the *HLA-A* locus corresponded to the classical allele HLA-A*02:01, which has also been implicated in multiple sclerosis^36^; however, while this allele is protective in multiple sclerosis, it increases the risk of AS. Previous studies have hinted at *HLA-DPB1* associations with AS, which we have confirmed here. HLA-DPB1, in conjunction with HLA-DPA1, forms the HLA-DP heterodimer, which typically plays a role in the presentation of exogenously derived peptides, such as microbial peptides, to CD4^+^ T lymphocytes. The strongest association was found with an amino-acid position located in the base of the peptide-binding groove of HLA-DP, suggesting that this polymorphism might impact on the peptide repertoire presented by HLA-DP.

Previous findings that *ERAP1* variants influence risk of disease in HLA-B*27 positive, but not negative individuals, strongly support the notion that both these molecules act in the same biological pathway to affect disease susceptibility^7^. We have now shown that HLA-B*40:01 interacts with *ERAP1* variants in the same manner. Similar genetic interactions involving *ERAP1* have been observed in two other immune-mediated disorders—psoriasis with *HLA-Cw6* (ref. 37) and Behçet’s syndrome with HLA-B*51 (ref. 38), two disorders that are already known to share genetic susceptibility factors with AS. It is likely that the similar molecular mechanisms are involved in these disorders, and that these include the pathways of MHC class I antigen presentation. To our knowledge, there is no evidence that HLA-B40, HLA-B51 or HLA-Cw6 have non-canonical disease-related properties such as those by which HLA-B27 is proposed to function in the pathogenesis of AS.

Analysis of polymorphic amino-acid positions in these AS-associated HLA molecules showed that the SNPs with the strongest evidence of association at each of these three loci were highly associated with amino-acid positions located in the peptide-binding groove of these proteins. From these results, we infer that antigen presentation to both CD4^+^ and CD8^+^ T lymphocytes is likely to be important in the pathogenesis of AS and/or its tissue specificity, although other mechanisms underlying the associations cannot formally be excluded.

MHC class I molecules contain six specificity pockets in the peptide-binding groove, alphabetically named A to F, which serve to anchor particular side chains of the bound peptide^39^. Position 97 of HLA-B is located in the C/F pocket, also referred to as the C-terminal pocket, which anchors the side chain of the C-terminal peptide residue^29^. Experimental evidence suggests that this position is important for protein function and shaping the peptide repertoire presented by HLA-B. Mutagenesis experiments have shown that Asn97 is important for HLA-B*27:04 surface expression; mutating this residue from Asn97 to Asp97 results in reduced surface expression and increased accumulation of unfolded protein in the ER, as well as reduced homodimers formation^40^; thus, Asn97 relative to Asp97 reduces ER stress and B27 homodimer formation, yet is associated with AS risk. Moreover, work in the mouse homologue has shown that changing residue 97 (W97R) results in altered peptide specificity and affinity for β_2_-microglobulin^41^, and previous crystallographic studies of viral peptides bound to HLA-B27 have shown that this position influences the location of the peptide in the binding groove of the molecule^42^. Last, this position was also found to be associated with HIV-1 viral control, where Val97 was found to provide the strongest protective effect to progression to AIDS, hypothesized to be through a mechanism of peptide presentation^43^. Asp97 is not shared by the AS-associated subtype HLA-B*27:07, where it is substituted by serine. Serine is also a polar amino acid and the substitution would be expected to have only minor effects on the protein structure. While AS is known to occur in individuals carrying HLA-B*27:07, its relative strength of association compared with other AS-associated HLA-B*27 subtypes is unknown.

In summary, with high-density genotyping of the MHC, we have demonstrated independent association signals located in HLA class I and II loci. Imputation of amino-acid residues in the classical HLA class I and II proteins resolved the peak of association at each of these loci to an amino-acid residue located in the peptide-binding groove of these proteins. Refining this analysis by imputation of classical HLA alleles showed that there are multiple risk and protective haplotypes in the *HLA-B* locus. Further, epistatic interaction was demonstrated between *ERAP1* and the HLA class I alleles HLA-B*27 and HLA-B*40:01.

## Methods

### Sample collection

All cases met the modified New York classification criteria for AS^44^. Nine thousand sixty-nine cases and 13,578 controls were recruited through a multi-center study coordinated by the International Genetics of Ankylosing Spondylitis Consortium^8^, and all samples were unrelated and met European ancestry criteria as detailed therein. All subjects provided written informed consent and the study was approved by the Princess Alexandra Hospital Research Ethics Committee (reference HREC/05/QPAH/221) and University of Queensland Research Ethics Committee (Project Clearance No: 2006000102). All samples were genotyped with the Illumina (San Diego, CA, USA) Infinium platform Immunochip^45^, and the current study was restricted to 7,264 markers in the MHC (chromosome 6, bps 29,602,876–33,268,403, NCBI Build 36 human genome coordinates).

### Imputation

We imputed SNPs across the MHC, and classical HLA class I and II alleles (*HLA-A*, *HLA-C*, *HLA-B*, *HLA-DRBI*, *HLA-DQA1*, *HLA-DQB1*, *HLA-DPA1* and *HLA-DPB1*) and their corresponding amino acids determinants with SNP2HLA^28^. Samples with cumulative dosage above 2.5, across all four-digit alleles for any one of the HLA loci, were removed from the analysis. SNPs, alleles or amino-acid residues were excluded from the analysis if the *r*^2^ imputation quality score was below 0.2.

Classical allele imputation at the *HLA-B* locus resulted in high-quality data, with a median sensitivity and specificity for imputed *HLA-B* alleles of 0.958 and 0.998, respectively (Supplementary Fig. 1). With our imputation strategy, similar imputation performance has previously been shown for the other HLA class I and II loci (*HLA-A*, *HLA-C*, *HLA-DRB1*, *HLA-DQA1*, *HLA-DQB1*, *HLA-DPA1* and *HLA-DPB1*), suggesting that imputation performance for these loci was also accurate in our study^28^,^43^,^46^,^47^.

### Statistical framework for association analysis

Associations of SNPs, HLA protein amino-acid positions and non-*HLA-B* alleles across the MHC locus were assessed with logistic regression, assuming an additive risk effect on the log-odds scale. To account for population stratification, we included as covariates 10 principal components for each individual, computed with 16,145 unlinked autosomal, non-MHC, SNPs with the tool shellfish ( http://www.stats.ox.ac.uk/~davison/software/shellfish/shellfish.php). The omnibus association test compares, via likelihood ratio test, the null model *H*_0_, where there is no risk effect at the position tested, against the alternative model *H*_1_, where the risk effect at the position is included in the model as a fixed effect:




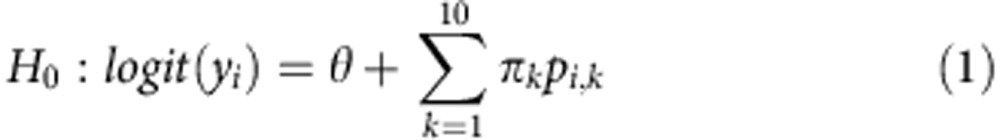







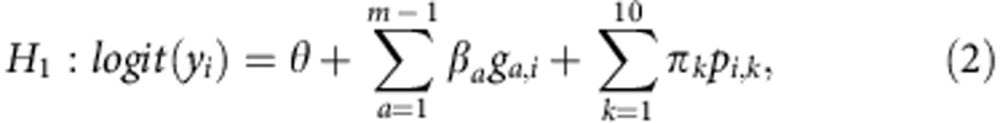




where *y*_*i*_ denotes the binary phenotype code for individual *i* (0=control and 1=case). The *π*_*k*_ parameter is the effect associated with each of the principal components and *p*_*i,k*_ is the value of the *k*th principal component for individual *i* . The *θ* parameter represents the sampling fraction (that is, the logistic regression intercept). In the alternative model, *a* indicates the specific allele being tested and *g*_*a,i*_ is the dosage (imputed or genotyped) of allele *a* in individual *i*. The *β*_*a*_ parameter represents the effect on the log odds of disease per allele. For testing a multi-allelic locus, nucleic or amino-acid positions, with *m* possible alleles we included *m*-1 *β* parameters, one for each allele, where the most common allele was selected as the reference allele. The likelihood ratio test that compares model *H*_0_ with *H*_1_ results in a test statistic that is *χ*^2^ distributed with *m*-1 degrees of freedom.

When testing for association with imputed classical HLA alleles, we defined a series of binary markers coding the presence or absence of the allele being tested, and each different allele was tested as a biallelic position as described above.

To identify independent effects. we performed conditional logistic regression by including the most strongly associated position/polymorphism as a fixed effect in both the null model *H*_0_ and the alternative model *H*_1_. We then analysed all positions as described above. Conditional analysis was repeated in an iterative fashion by sequentially adding the most significant positions as fixed effects until no significant position or polymorphism was observed. Allelic associations were deemed significant with *P*<10^−5^, this statistical significance threshold accounted for 5,000 independent tests using Bonferroni correction. Two tests were considered independent if the two SNPs had a pairwise correlation (*r*^2^)<0.90, which resulted in 3,252 SNPs independent tests. For the special case of *HLA-B* alleles where we had a higher prior probability of association, we defined significance as *P*<10^−3^ as only 38 alleles were tested.

## Additional information

**How to cite this article**: Cortes, A. *et al*. Major histocompatibility complex associations of ankylosing spondylitis are complex and involve further epistasis with ERAP1. *Nat. Commun.* 6:7146 doi: 10.1038/ncomms8146 (2015).

## Supplementary Material

Supplementary Information Supplementary Figure 1 and Supplementary Tables 1-8

## Figures and Tables

**Table 1:** Most significant polymorphic positions (omnibus test) and imputed classical alleles associated with ankylosing spondylitis susceptibility (*P*-value<1 × 10^−2000^).

**Position**	**rs**	**AA position**	**Classical allele**	***χ*** ^**2**^	**DF**	***P*** **-value**
31,432,180	—	97	—	14,857	5	<10^−3,221^
31,432,180	rs1071652	—	—	14,841	3	<10^−3,221^
31,430,829	rs41558317	—	—	14,823	1	<10^−3,221^
31,432,179	rs1140412	—	—	14,823	2	<10^−3,219^
—	—	—	HLA-B*27	14,820	1	<10^−3,221^
31,432,506	—	70	—	14,812	3	<10^−3,215^
31,432,129	—	114	—	14,402	2	<10^−3,128^
31,432,130	rs709055	—	—	14,401	2	<10^−3,128^
31,432,131	rs1050628	—	—	14,389	1	<10^−3,127^
—	—	—	HLA-B*27:05	14,220	1	<10^−3,090^
31,430,834	rs3819282	—	—	13,798	1	<10^−2,999^
31,430,345	rs3819299	—	—	13,757	1	<10^−2,990^
31,430,346	rs3819299	—	—	13,757	1	<10^−2,990^
31,451,646	rs4463302	—	—	12,898	1	<10^−2,803^
31,432,485	—	77	—	12,871	2	<10^−2,795^
31,432,486	rs1131217	—	—	12,849	1	<10^−2,793^
31,377,108	rs2394967	—	—	11,613	1	<10^−2,524^
31,381,125	rs6905036	—	—	11,552	1	<10^−2,511^
31,432,208	rs41556113	—	—	10,929	1	<10^−2,376^
31,432,843	rs41553720	—	—	10,299	2	<10^−2,237^
31,432,515	—	67	—	9,741	4	<10^−2,112^
31,432,515	rs1071816	—	—	9,725	3	<10^−2,110^
31,518,387	rs2844510	—	—	9,525	1	<10^−2,071^

AA, amino acid; DF, degrees of freedom.

**Table 2:** Evidence for association of *HLA-B* alleles with susceptibility to ankylosing spondylitis. Effect sizes and levels of significance were estimated in stepwise conditional procedure, where for rounds 2 and onwards the test conditioned on the previous alleles.

**Round**	***HLA-B*** **allele**	**OR (95% CI)**	***P*** **-value**
1	*27:05*	62.41 (56.90–68.45)	<10^−321^
2	*27:02*	43.41 (29.80–63.23)	1.07 × 10^−122^
3	*07:02*	0.82 (0.74–0.91)	5.04 × 10^−6^
4	*57:01*	0.75 (0.61–0.92)	5.13 × 10^−4^
5	*51:01*	1.33 (1.14–1.56)	2.14 × 10^−3^
6	*47:01*	2.35 (1.43–3.86)	2.25 × 10^−3^
7	*40:02*	1.59 (1.19–2.14)	4.65 × 10^−3^
8	*13:02*	1.43 (1.14–1.80)	4.29 × 10^−3^
9	*40:01*	1.22 (1.06–1.40)	4.93 × 10^−3^
All other alleles			>0.05

CI, confidence interval; OR, odds ratio;

**Table 3:**
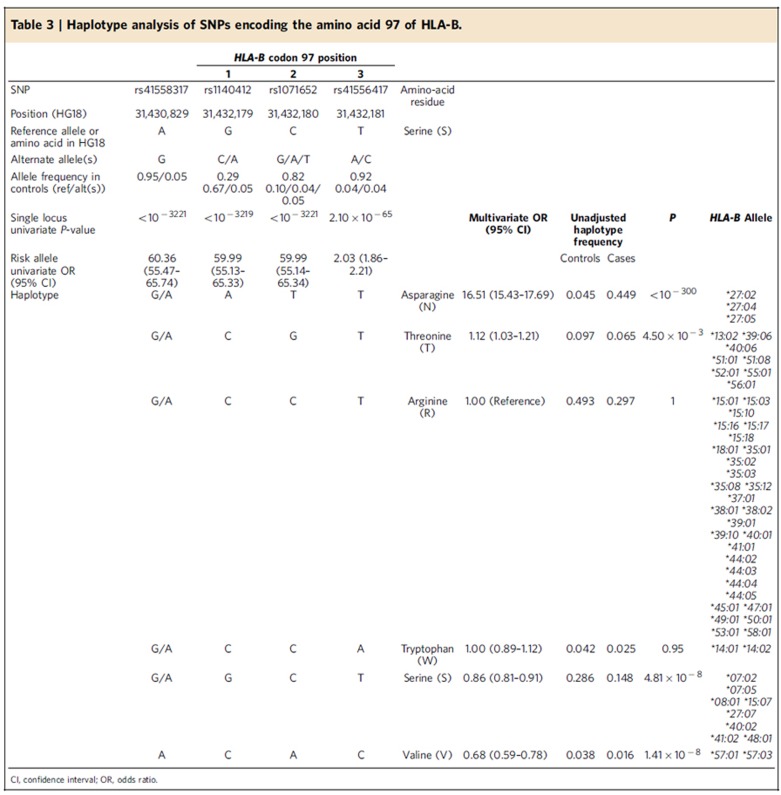
Haplotype analysis of SNPs encoding the amino acid 97 of HLA-B.
