# Supplementary material for: Major histocompatibility complex associations of ankylosing spondylitis are complex and involve further epistasis with ERAP1
Source: Nat Commun. 2015 May 21;6:7146. doi: 10.1038/ncomms8146 (PMC4443427; doi:10.1038/ncomms8146)
Supplement: Supplementary Information — Supplementary Figure 1 and Supplementary Tables 1-8 [file ncomms8146-s1.pdf]

Supplementary Figures

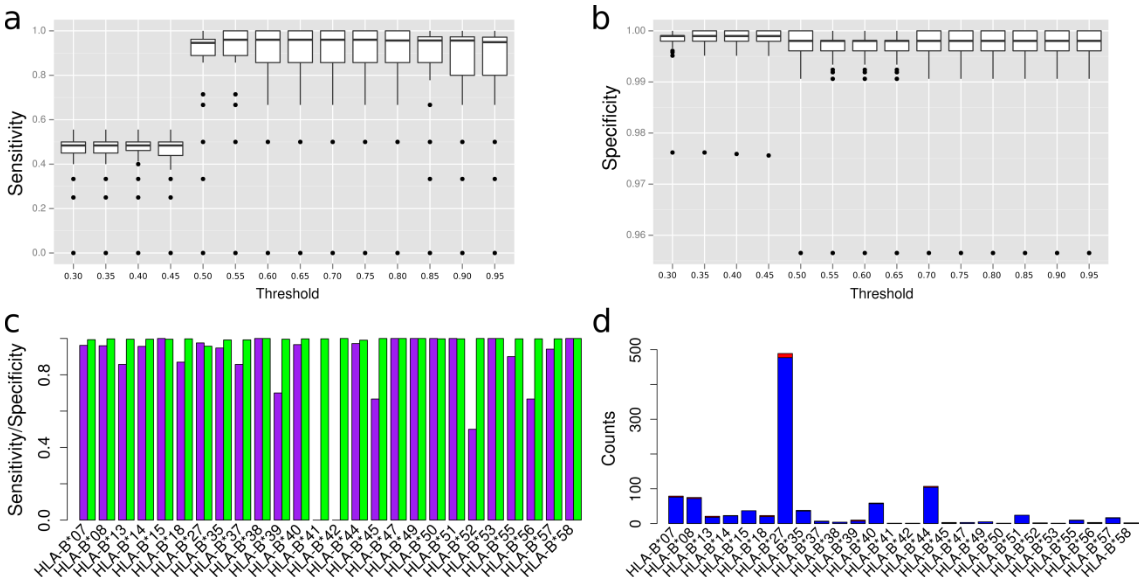

Supplementary Figure 1 Performance of classical allele imputation at two digit resolution in the *HLA-B* locus. HLA typing at the *HLA-B* locus was available for 536 AS patients, which also had imputed genotype data. The optimal threshold dosage for genotype calling was assessed by computing the sensitivity (a) and specificity (b) for all HLA-B alleles at different dosage thresholds. Error bars represent 95% confidence intervals. For a dosage threshold of 0.7 the median sensitivity (c; purple bars) and specificity (c; green bars) for all HLA-B alleles was 0.958 and 0.998, respectively. (d) Proportion of false negatives in the imputed alleles for each allele at a 0.7 dosage threshold, blue sections are proportional to the number of true positives and red sections are proportional to the number of false negatives.

Supplementary Tables

Supplementary Table 1 No evidence of interaction between *HLA-B*27* and *HLA-B*40* risk alleles. Odds of disease for each genotype class was computed against the reference (*HLA-B*27*-negative and **40*-negative). The risk effect for the genotype $HLA\text{-}B*27^{-}/HLA\text{-}B*40^{+}$ was estimated to be 1.35 and for the class $HLA\text{-}B*27^{+}/HLA\text{-}B*40^{-}$ the effect was 47.76. Under no interaction between these two alleles we would expect the effect size of the genotype class $HLA\text{-}B*27^{+}/HLA\text{-}B*40^{+}$ to be 1.35*47.76 = 64.48 (assuming a model for risk on the multiplicative scale), which is close to what we observed, 65.19.

Locus Genotypes	Counts		OR	CI (95%)	*P*-value
	Controls	Cases			
HLA-B*27/ HLA-B*27	33	363	84.78	58.9-125.93	3.23×10^{-279}
HLA-B*27/ HLA-B*40	97	822	65.19	52.42-81.85	$< 1 \times 10^{-300}$
HLA-B*27/non-HLA-B*40	1,069	6,626	47.76	43.8-52.14	$< 1 \times 10^{-300}$
HLA-B*40/ HLA-B*40	61	12	1.52	0.74-2.85	0.20
HLA-B*40/non-HLA-B*27	1,587	278	1.35	1.17-1.55	3.38×10^{-5}
other	10,723	1,391	1.00	Reference	

Supplementary Table 2 Association results in groups with different genotypes for the *HLA-B*27* and *–B*07* alleles.

Locus Genotypes	Counts		OR	CI (95%)	*P*-value
	Controls	Cases			
HLA-B*27/ HLA-B*27	32	353	99.89	69.01-149.63	1.16×10^{-287}
HLA-B*27/ HLA-B*07	174	886	46.09	38.63-55.28	$< 10^{-300}$
HLA-B*27/non-HLA-B*07	963	6,324	59.38	54.12-65.66	$< 10^{-300}$
HLA-B*07/ HLA-B*07	285	15	0.48	0.26-0.8	2.97×10^{-3}
HLA-B*07/non-HLA-B*27	2,928	244	0.75	0.65-0.87	1.35×10^{-4}
other	8,876	980	1.00	Reference	

Supplementary Table 3 Association results in groups with different genotypes for the *HLA-B*27* and *–B*51* alleles.

Locus Genotypes	Counts		OR	CI (95%)	*P*-value
	Controls	Cases			
HLA-B*27/ HLA-B*27	32	353	112.13	77.41-166.8	3.80×10^{-305}
HLA-B*27/ HLA-B*51	50	379	76.92	56.71-106.41	2.48×10^{-311}
HLA-B*27/non-HLA-B*51	1,087	6,830	63.74	58.27-69.82	$< 10^{-300}$
HLA-B*51/ HLA-B*51	24	5	2.12	0.63-5.68	0.18
HLA-B*51/non-HLA-B*27	1,008	146	1.47	1.22-1.77	8.11×10^{-05}
other	11,039	1,086	1.00	Reference	

Supplementary Table 4 Association results in groups with different genotypes for the *HLA-B*27* and *–B*57* alleles.

Locus Genotypes	Counts		OR	CI (95%)	*P*-value
	Controls	Cases			
HLA-B*27/ HLA-B*27	32	353	106.37	73.44-159.37	4.15×10^{-299}
HLA-B*27/ HLA-B*57	53	188	34.18	24.91-47.55	8.92×10^{-137}
HLA-B*27/non-HLA-B*57	1,083	7,025	62.46	57.13-68.26	$< 10^{-300}$
HLA-B*57/ HLA-B*57	14	1	0.69	0.02-4.54	1
HLA-B*57/non-HLA-B*27	923	81	0.85	0.66-1.07	0.17
other	11,157	1,157	1.00	Reference	

Supplementary Table 5 Association results in groups with different genotypes for the *HLA-B*27* and *–B*47* alleles.

Locus Genotypes	Counts		OR	CI (95%)	*P*-value
	Controls	Cases			
HLA-B*27/ HLA-B*27	32	353	108.41	75.01-161.11	1.34×10^{-302}
HLA-B*27/ HLA-B*47	1	25	245.63	40.1-9417.17	4.12×10^{-25}
HLA-B*27/non-HLA-B*47	1,136	7,187	62.09	57.09-67.95	$< 10^{-300}$
HLA-B*47/ HLA-B*47	0	0	NA	NA	NA
HLA-B*47/non-HLA-B*27	77	17	2.17	1.2-3.72	6.65×10^{-3}
other	12,019	1,223	1.00	Reference	

Supplementary Table 6 Association results in groups with different genotypes for the *HLA-B*27* and *–B*13* alleles.

Locus Genotypes	Counts		OR	CI (95%)	*P*-value
	Controls	Cases			
HLA-B*27/ HLA-B*27	32	353	108.92	75.38-161.96	1.41×10^{-302}
HLA-B*27/ HLA-B*13	9	141	154.59	78.83-346.28	3.91×10^{-130}
HLA-B*27/non-HLA-B*13	1,128	7,072	61.81	56.77-67.72	0
HLA-B*13/ HLA-B*13	3	1	3.29	0.06-41.03	0.32
HLA-B*13/non-HLA-B*27	491	64	1.29	0.97-1.69	0.07
other	11,602	1,175	1.00	Reference	

Supplementary Table 7 Level of conditional association significance at polymorphisms associated with *HLA-B*27*. For each type of polymorphism (SNP, amino acid residue and position, and *HLA-B*27* at two digit resolution imputation and *HLA-B*27:05* at four digit resolution imputation) we performed pairwise conditional analyses. After conditioning on position 97 we observed level of significance at $p>10^{-4}$ for the other polymorphisms, while conditioning on any of the other polymorphisms resulted in strong significance at position 97 ($p<5x10^{-10}$).

		Association Test				
		rs41558317	HLA-B a.a. position 97 – N dosage	HLA-B a.a. position 97 – omnibus test	*HLA-B*27*	*HLA-B*27:05*
Conditional polymorphism	rs41558317	--	0.42	$4.63x10^{-10}$	$6.78x10^{-3}$	0.18
	HLA-B a.a. position 97 – N dosage	$1.00x10^{-3}$	--	$1.44x10^{-10}$	$2.56x10^{-4}$	0.09
	HLA-B a.a. position 97 – omnibus test	$1.15x10^{-3}$	1.00	--	$2.27x10^{-4}$	$7.75x10^{-2}$
	*HLA-B*27*	0.01	0.11	$1.66x10^{-10}$	--	0.05
	*HLA-B*27:05*	$8.57x10^{-128}$	$8.35x10^{-126}$	$4.67x10^{-132}$	$2.14x10^{-128}$	--

Supplementary Table 8 Level of conditional association significance at the five most significant amino acid positions in the HLA-B protein. For each position we conditioned on it and tested for association at the other four positions.

		Omnibus amino acid tests					
		97	70	114	77	67	9
Conditional amino acid position	97	--	0.014	1.08×10^{-3}	2.28×10^{-4}	0.040	0.016
	70	6.28×10^{-11}	--	6.84×10^{-5}	6.05×10^{-3}	0.882	7.28×10^{-3}
	114	3.46×10^{-99}	1.32×10^{-92}	--	2.10×10^{-9}	4.16×10^{-17}	1.12×10^{-13}
	77	$< 1 \times 10^{-300}$	$< 1 \times 10^{-300}$	$< 1 \times 10^{-300}$	--	1.21×10^{-144}	1.32×10^{-33}
	67	$< 1 \times 10^{-300}$	$< 1 \times 10^{-300}$	$< 1 \times 10^{-300}$	$< 1 \times 10^{-300}$	--	$< 1 \times 10^{-300}$
	9	$< 1 \times 10^{-300}$	$< 1 \times 10^{-300}$	$< 1 \times 10^{-300}$	$< 1 \times 10^{-300}$	$< 1 \times 10^{-300}$	--
